# Psychotherapy in historical perspective

**DOI:** 10.1177/0952695117703243

**Published:** 2017-04-20

**Authors:** Sarah Marks

**Affiliations:** Birkbeck, University of London, UK

**Keywords:** clinical epistemologies, historiography, history of the psy-disciplines, psychotherapy, therapeutic relationship

## Abstract

This article will briefly explore some of the ways in which the past has been used as a means to talk about psychotherapy as a practice and as a profession, its impact on individuals and society, and the ethical debates at stake. It will show how, despite the multiple and competing claims about psychotherapy’s history and its meanings, historians themselves have, to a large degree, not attended to the intellectual and cultural development of many therapeutic approaches. This absence has the potential consequence of implying that therapies have emerged as value-free techniques, outside of a social, economic and political context. The relative neglect of psychotherapy, by contrast with the attention historians have paid to other professions, particularly psychiatry, has also underplayed its societal impact. This article will foreground some of the instances where psychotherapy has become an object of emerging historical interest, including the new research that forms the substance of this special issue of *History of the Human Sciences*.

## Introduction

Psychotherapy has been cast in various lights. For some, it is an age-old method of healing, the ‘care of the soul’ that harks back, at the very least, to ancient Greece. To others, it is a product of a particularly modern moment at the end of the 18th century, linked with social coercion and the rise of the bourgeois family, which later ascended to become a prime technology of autonomy and self-regulation without which liberal democracies might not function. To some, it has provided modes of describing personal experience that have created fundamentally new ways of conceiving of the self, or, indeed, of *being*. For others still, it is merely a form of religious practice camouflaged by the language of medical science. This article will briefly explore some of the ways in which the past has been used as a means to talk about psychotherapy as a practice and as a profession, its impact on individuals and society, and the ethical debates at stake. It will show how, despite these multiple and competing claims about psychotherapy’s history and its meanings, historians themselves have, to a large degree, not attended to the intellectual and cultural development of many therapeutic approaches. This absence has the potential consequence of implying that therapies have emerged as value-free techniques, outside of a social, economic and political context. The relative neglect of psychotherapy, by contrast with the attention historians have paid to other professions, particularly psychiatry, has also underplayed its societal impact. This article will foreground some of the instances where psychotherapy *has* become an object of emerging historical interest, including the new research that forms the substance of this special issue of *History of the Human Sciences*.

Psychotherapy certainly has come to play a role in the lives of many individuals in the contemporary western world since the term’s conception in the late 19th century (Carroy, 2000: 11; Shamdasani, 2005: 1). In 2004, survey data published by *Psychology Today* indicated that 30 million American citizens, approximately 14.3% of the population, had accessed therapy within the previous two years (Howes, 2008).^1^ Estimates made at the turn of the 21st century by the American Psychological Association suggested that there were between 200,000 and 250,000 psychotherapists in the USA, along with 100,000 professional counsellors, and 50,000 marriage and family therapists. By contrast, there were only 41,000 psychiatrists in the country (DeLeon, Patrick, Kenkel and Garcia-Shelton, 2011: 51). In Britain, the National Health Service has funded and promoted an initiative for Increasing Access to Psychological Therapies since 2008, largely justified by political arguments about improving national economic performance (Marks, 2012). Psychotherapy and those who practise it have a stake in contemporary societies, in both the public and private spheres. But how did these interventions develop? What were their intellectual origins, and what were the institutional and cultural forces that shaped them? Have different types of approach existed within different types of society, or during different periods of history, and how have these interacted with wider political and social debates? Furthermore, how have the therapeutic professions created new categories of personhood in the shape of the ‘patient’, ‘client’, or ‘service-user’, and with what effects? For many psychotherapies, these historical questions remain wide open.

Psychoanalysis as a particular mode of psychotherapeutic intervention, to be sure, has its own sophisticated and ever-burgeoning historiography.^2^ Yet this is still not the case for many of the approaches to treatment that may owe a debt to a psychoanalytic worldview, but have branched off in assorted directions. The popular humanistic psychotherapy of Carl Rogers is one of the most striking examples of such a lacuna.^3^ Approaches that have departed dramatically from psychoanalysis have still, to a large degree, eluded historical interrogation. As a result, their origins and development – both intellectual and institutional – remain opaque to clients and service-users, not to mention many of their own practitioners. The obscuring of such histories – however accidental or unintentional this may be – has ethical ramifications in terms of transparency. Given the authority that psychotherapeutic knowledge has come to hold, particularly when bolstered by state services and government support, there are also political implications as a result of this neglect.

There is thus an imperative to reflect on the emergence of different modes of therapy, and the modifications to these approaches during the course of their development. Widening the angle of the lens to take in a historical perspective uncovers how they often came to be shaped by factors outside of the consulting room. It raises epistemological questions about how some psychotherapies have located themselves in proximity to claims about ‘scientificity’, or ‘evidence base’, and the way the criteria for such claims has changed since the 19th century. It also brings to the fore the plurality of assumptions embedded within different approaches, in terms of models of mind and human culture (Shamdasani, 2017: 367).

The breadth of practices that had become associated with psychotherapy by the end of the 20th century has indeed struck even some within the profession as alarming. For the Czech-American psychiatrist Jan Ehrenwald, writing an introduction to a large textbook aimed at clinicians in 1991, psychotherapy appeared to be

…headed in all directions at once. Freudian analysis is challenged by gestalt therapy, transactional analysis, screaming cures, nude marathons; the proliferation of diverse ‘pop’ therapies…Some proclaim the dawn of an age of do-it-yourself mind-control through biofeedback, transcendental meditation…(Ehrenwald, 1991: 5)

Yet since the 1980s there has also been a rise in the use of terms such as ‘integration’ and ‘eclecticism’, particularly in British and American psychotherapy (Dryden, 1992; Gold, 1996; Norcross and Goldfried, 2005; Palmer and Woolfe, 1999). On the one hand, these indicate a certain pragmatism: an effort to transcend dogmatic adherence to particular traditions or identities, and to draw on a wide theoretical base as appropriate for the needs of the client. The move towards integration can be seen not only in ‘integrative therapy’ itself, but also in the twinning of cognitive and behavioural approaches into cognitive behavioural therapy (CBT) and, in turn, CBT’s subsequent polyvalent merging with multiple other schools, during its so-called ‘third wave’ (Marks, 2012; Rachman, 1986). These hybrids have included diverse practices such as Mindfulness meditation, the psychodynamically inspired Schema Therapy approach, through to Eye Movement Desensitization and Reprocessing (ibid.: 17–18).

Such syntheses should not come as a surprise, given the contemporaneous shift towards interdisciplinarity in wider fields of social science, medicine and the humanities. Nevertheless, it raises questions about the consequences of gathering competing understandings of mind and behaviour under a single rubric. While many clinicians whose identities are inextricably linked to specific schools are unlikely to participate in such integration, the shift towards dialogue between different approaches since the 1980s does hint at something of an unprecedented professional rapprochement in certain quarters. This contrasts starkly with the vehement clashes of the 1950s between, say, psychoanalysis and behaviourism, or indeed the feuds within the psychoanalytic movement itself prior to this (Buchanan, 2010: ch. 6; Falzeder, 2015; King and Steiner, 1991). The umbrella term ‘psychotherapy’ may now provide shelter for a plurality of practitioners who are more inclined to coexist, and even collaborate, with others who differ in worldview but share the same professional title. But ‘psychotherapy’, as a result, also acts as a gloss, which effectively conceals ontological incongruities and historical conflicts.

The contributions to this special issue focus on Anglo-American and European debates in the history of psychotherapy.^4^ They open up a range of histories, from the very early use of the term psycho-therapeutic in late-Victorian England, to debates surrounding the therapeutic relationship, or the ethics of behaviour modification, that are still very much alive today. The articles variously bring to light forgotten figures, clinical techniques and theories of mental health and disorder. A number of authors explore the conflicts and controversies within professional groups, or between clinicians and their target client groups. Others examine the ways in which psychotherapeutic knowledge and practice came to have a voice in wider political and cultural debates, and vice versa.

## Historiographies

Before introducing the new articles in the special issue, this section will map the terrain of the literature to which they are contributing. How has the history of psychotherapy been represented, and for what purposes? While historians’ engagement with psychotherapy has been somewhat scattered, clinicians themselves have made enthusiastic use of history as a way to reflect on the institutional and theoretical developments of their approaches, and at times to legitimate and celebrate their profession (Bankart, 1997; Dryden, 1996; Ehrenwald, 1991; Hall, Pilgrim and Turpin, 2015; Moss, 1999; Norcross, Van den Bos and Freedheim, 2011; Schmidbauer, 1998; Zeig, 1991).

One popular strategy of validation overlooks the modern invention of the term ‘psychotherapy’ altogether, and traces a certain therapeutic sensibility directly back to ancient Greece (Bankart, 1997; Jackson, 1999; Schmidbauer, 1998). Any suggestion of equivalence between classical thought and practice and contemporary approaches raises obvious problems of anachronism, but it does hint at something of genuine significance, namely the active appropriation of classical motifs within the foundational texts of a number of modes of therapy. This is the case for psychoanalysis itself, with Freud’s dialogue with Greek philosophy explicated in the work of Alfred Tauber (2010). The Jungian analyst Carl Meier drew more explicit parallels between analytic psychology and the healing traditions of the temple of Asclepius, looking back to the use of isolation within the temple, along with the promotion of sleep to bring about therapeutic and enlightening dreams. Meier also pointed out the analogy between the wound of Chiron the centaur, the figure associated with healing in Greek mythology, and the Jungian image of the analyst as wounded healer (Meier, 1967[1949]; Samuels, 1985: 187).

The cognitive-behavioural traditions have also situated themselves within Greek – and more specifically Stoical – thought (Evans, 2012). Windy Dryden and Arthur Still have traced what they call ‘the legacy of Epictetus’ in the Rational Emotive Behaviour Therapy (REBT) of Albert Ellis, showing how both he and Aaron Beck looked back to the maxim ‘Men are disturbed not by things, but by the views which they take of things’, as the origin of their own practice (Dryden and Still, 2012: xv).^5^ They argue further that Ellis’s position was not merely analogous, but homologous with Epictetus’, demonstrating his ‘active seeking of resources’ in Stoical writings, which enabled his turn away from psychoanalysis by offering a competing framework from which to draw (ibid.: 41–2). Dryden and Still assert that the acceptance of Ellis’s REBT in the mid-century was facilitated by a contemporaneous flourishing of religious and therapeutically orientated texts which themselves drew on Stoicism, ‘rooted in the post-Enlightenment urge to put the world to rights and restore the American Dream through personal and political transformation’ (ibid.: 41). The thriving market for self-help books, such as Dale Carnegie’s 1948 *How to Stop Worrying and Start Living* (1948), peppered with quotations from Marcus Aurelius, was a prime example of such a sensibility: the popularity of which Ellis was able to build on to consolidate his new brand of psychotherapy (Dryden and Still, 2012: 41).

At another remove, the psychoanalytic psychotherapist Laurence Spurling has sought to critically understand the way in which therapists look to their own profession’s past for inspiration, and often prize the ‘traditions’ in which they situate themselves (Spurling, 1993). Drawing a parallel with Talmudic scholarship, Spurling notes that the pages of any psychotherapeutic journal will contain countless examples of ‘contributors [who] take pains to place themselves in a line of thinking or practice which goes back to a recognized figure…and often back to the founding figures of Freud or Jung…with an overwhelming priority placed on knowledge of and respect for what has come before’ (ibid.: 5). While histories provide a vast resource for creative innovation in theory and technique, these traditions also have the potential to foster a culture of conservatism and deference.

Beyond the clinical professions, authors who have been overtly critical of the psy-disciplines, casting them as normalizing forces, have also sought out origin myths that pre-date the modern coinage of ‘psychotherapy’ itself. For Thomas Szasz, writing in his 1978 book *The Myth of Psychotherapy* (a follow-up to his *The Myth of Mental Illness* (1961), which became a canonical text for the so-called ‘anti-psychiatry’ movement), therapy was an example of how ‘coercion and conversation became analogized to medical treatment’ (Szasz, 1988[1978]: xii). The medicalized formulation of psychotherapy concealed its true roots, which, he argued, grew out of a combination of rhetoric and religion, going back to early- and pre-Hellenic healing practices, later taken up in other religious traditions as a form of pastoral cure of the soul (ibid.: ch. 3).

Michel Foucault, in what has perhaps become the most well-known critique of psychiatric and therapeutic interventions, identified a shift in the way western society conceptualized madness with the establishment of ‘moral treatment’ at the end of the 18th century at the York Retreat in England under the Tuke family, and with Phillipe Pinel’s work at La Salpêtrière and Bicêtre hospitals in Paris (Foucault, 2006a[1972], 2006b). A change in perspective came about at this modern moment, away from a sole focus on confinement and restraint and towards a ‘benevolent’ attitude towards the insane which sought out the remnants of their reason in order to effect their restoration, in part through the simulation of a family setting within the asylum. Foucault himself queried the benevolence of the new approach. He asserted that the power relationships that played out between asylum staff and their patients inherently involved judgement and punishment, and that practices such as therapeutic work were just as coercive as physical restraint (Foucault, 2006a[1972]: 485). The familial metaphor masked such power behind a veneer of kindness, and ultimately acted in the interests of societal norms. ‘From this point onwards’, Foucault argued, ‘and for a period whose end is still impossible to see, the discourses of unreason became inextricably linked to the half-real, half-imaginary dialectic of the family’ (ibid.: 490).^6^ The Foucaldian torch has latterly been taken up by sociologist Nikolas Rose, now one of the most oft cited commentators on the rise of the ‘psy’ disciplines in the 20th century (Rose, 1991; 1998). Rose has argued that psychotherapy functions as one of the ‘technologies of the self’ that have become crucial to liberal democratic societies, as they allow for the self-regulation of free citizens, enabling ‘competent autonomous selfhood’ at an individual level, which in turn sustains the functioning of the polity (Rose, 1998: 100).

Another way of considering the interaction between psychotherapy and society has come from the concept of ‘looping’. Ian Hacking’s 1995 essay, ‘The Looping Effect of Human Kinds’, outlined the manner in which the social and human sciences – including the ‘psy’ professions – have created categories of person, behaviour or disorder, such as ‘child abuse’ or ‘multiple personality disorder’, with a view to knowing ‘how people of a kind will respond to attempts to help them or to modify their behavior…Groups of experts now collaborate to say that together they are members of the “helping professions”: social workers, therapists [etc.]…’ (Hacking, 1995: 360). Categories such as ‘multiple personality disorder’, for Hacking, can be designated as ‘human kinds’ – as opposed to the ‘natural kinds’ of the physical and biological sciences. What distinguishes them from the latter is the potential they have to

…enable us to redescribe our past to the extent that people can come to experience *new* pasts…To take an extreme example, some people come to see themselves as incest survivors, which in turn changes their lives and their relationships to their families. This is no mere matter of recovering forgotten trauma; it is a matter of there being new descriptions available…One of the more powerful words in this group of examples is ‘trauma’ itself, naming a relatively new kind of human experience…(Hacking, 1995: 368–9)

These new kinds and experiences, in turn, provoke further changes in expert practices and classifications, with new causal links being created between them: multiple personality disorder, for example, has become almost inextricably linked to child abuse (1995: 369). This has implications for the way in which theories of mental disorder are generated, but also for how clinicians conceptualize and interact with their clients. It has inflected the tasks of particular psychotherapies used in the treatment of such disorders by framing the process around the location of a particular traumatic event, or moment of abuse. By dealing in human kinds, the ‘psy’ professions therefore engage in what Hacking has called ‘making up people’ (Hacking, 2006: 23).

Discussing psychotherapeutic practices more specifically, the historian Sonu Shamdasani has also acknowledged the capacity they possess to create new modes of being:

In the twentieth century, psychotherapy has been an ontology-making practice. The therapeutic encounter became a site where individuals not only were cured, or not, as the case might be; but also learnt to articulate their suffering in new idioms, reconceive their lives according to particular narrative templates, and took on conceptions concerning the nature of the mind and reality. The consequence of this has been the generation of a plethora of optional ontologies. (Shamdasani, 2017: 367)

The dialectical process by which the popular reception of these therapeutic concepts went on to inform the way individuals presented themselves to therapists resulted in ‘a moebius strip of circulating feedback loops’ (Shamdasani, 2017: 375). This focus on the ‘optional’ nature of the ontologies generated by psychotherapy opens up the possibility for resistance, reminding us that clients can opt in, or can equally choose to reject them (my use of resistance here is taken in a political sense, rather than the psychoanalytic sense, although the latter obviously raises the fact that such choices can be cast by professionals as a symptom or defence).^7^ By this token, the circulating loop is not an inevitability, and individuals – or, in some cases, even groups – have sometimes taken the choice to sideline the ontologies proffered by ‘psy’ professions. In turn, they can generate alternative categories of selfhood, and practices by which to manage these. A clear example of such a process is exemplified in Patrick Kirkham’s article in this special issue, which explores autistic self-advocates’ objection to behavioural therapy interventions, as will be discussed below. Sarah Chaney’s recent work on the history of self-harm, written from a perspective of lived experience, has also queried the authority of professional expertise, including the way therapeutic approaches have framed the phenomenon of self-harm, and those who engage in it, in terms of defined categories that should be treated in specific ways. She has argued that while models of psychopathology may offer ‘a useful and potentially therapeutic’ way of approaching self-harm, they can fail by attempting to

…function *outside* of all other narratives, remaining separate from other ways of understanding…if there is one thing that the history of self-mutilation can teach us, it is that no one meaning of self-harm can be considered more ‘true’ or genuine than any other, and that medical, social and artistic solutions to mental distress can only function in conjunction with each other. (Chaney, 2017: 243)

Even with the rise of ‘co-production’ and ‘user engagement’ in contemporary clinical practice, histories such as these, which seek to understand – or are written from – the experience of the psychotherapeutic patient, client or service-user, remain a rarity (Foster, 2015).

Recently emerging literature has focused on the history of a diverse range of more specific therapeutic practices in their local setting. Key contributions have included Rachael Rosner’s work on Aaron Beck and the origins of cognitive therapy, Eva Illouz’s work on psychotherapeutic self-help, and Deborah Weinstein on the rise of family therapy in the USA (Rosner, 2014; Illouz, 2008; Weinstein, 2013). In the British context, Gavin Miller’s work has explored on the interrelationship between therapy and religion in Scotland, and Rhodri Hayward has charted the uses of psychotherapeutics in postwar British primary care (Miller, 2015; Hayward, 2014).^8^ This volume builds on this work by bringing to the fore new research, with contributions from emerging authors in the field.

## New contributions

This special issue is opened by Sarah Chaney’s exploration of the early uses of the term ‘psycho-therapeutic’ in the late 19th century by the English asylum alienist Daniel Hack Tuke (1827–95), shortly after its original coinage in continental Europe (Carroy, 2000: 11; Shamdasani, 2005: 1). Tuke, a descendant of the Quaker founders of moral therapy at the York Retreat, described the mechanism by which ‘the Imagination’ could be used as a mediator between either mind and body, or patient and doctor, for the purposes of treatment, with the relationship between the latter two emphasized as a key part of the process. Suggestion and hypnotism, both immensely popular techniques in the late Victorian period, were thought by Tuke to offer the possibility not only of treating mental symptoms, but also of healing the body’s physical ailments. Despite his significant publication record, Chaney’s article is the first to fully reconstruct Tuke’s thought, bringing to light an overlooked individual in the history of medicine and the psy-professions.

A number of articles in this issue revisit forgotten figures and practices, unearthing what these stories can tell us both about psychotherapeutics and their development, and also about the cultural and political surrounds of their age. David Freis writes about the remarkable invention of subordination–authority–relation (SAR) psychotherapy of Erwin Stransky (1877–1962), a practice that competed with psychoanalysis in inter-war Vienna. Freis argues that although SAR therapy had a relatively short shelf life, it nevertheless tells us about how those from the psychiatric community responded to the competition presented by psychoanalytic techniques within Freud’s own locale. The history of SAR also provides us with a study of the overt application of an individual’s political orientation to the shaping of his or her therapeutic techniques. Focusing on the treatment of neurosis, Stransky attempted to recreate his idealized system of a hierarchical, authoritarian society within the microcosm of the consulting room, aiming to bring about cure through the subordination of the patient. This unusual case of a therapy, designed specifically in order to serve a right-wing agenda, offers a counter-balance to the more frequently cited examples of therapies associated with a socialist or liberal *Weltanschauung* (Danto, 2000, 2005).

Martin Liebscher examines the transplantation of debates within the Jungian analytic community after emigration from Europe to Mandate Palestine in the 1930s and after. The article focuses on the key figure of Erich Neumann, a mentee of Carl Jung, and two analysts who, in Liebscher’s words, have been ‘forgotten or deleted from the historical record’: Margarete Braband-Isaac (1892–1986) and Max M. Stern (1895–1982). Liebscher’s research is itself a product of a larger-scale historiographical intervention into the history of psychotherapy, funded as it was by the Philemon Foundation (2017). The purpose of this independent, non-profit foundation has been to edit and prepare the works of Jung for publication and, as such, it addresses an imbalance in the scholarship hindered by the limited availability of Jungian texts, by contrast with the more established historiography of Freud and Freudian psychoanalysis.

A further émigré figure from interwar Europe rediscovered in this special issue is the Hungarian psychiatrist Francis Reitman, who resettled in the United Kingdom in 1938. Connor Cummings traces the development of his thought from his training in Budapest to his period at the Maudsley Hospital in London, and his eventual post as the research scientist attached to Adamson and Cunningham Dax’s famous art therapy studio at the Netherne Hospital in Surrey. Echoing a point made in Sarah Chaney’s article on Daniel Hack Tuke, Cummings shows that Reitman’s conception of mental disorder and its treatment, just as much as Tuke’s, eschewed clear binaries between somatic or biological explanations and psychological concepts. He drew instead on eclectic sources, from drug-induced coma treatments through to psychoanalysis. During his time at the Netherne, Reitman analysed the therapeutic artistic output of hundreds of patients, and used the results to inform his understanding of mental illness, elaborated in his popular books *Psychotic Art* (1950) and *Insanity, Art and Culture* (1954). Reitman’s work opens up the question of how psychotherapeutic practices themselves contribute to scientific theory-building about the nature of the mind and mental health. Cummings discusses the way in which a synthetic analysis of multiple cases enabled Reitman to make some broader assertions about the insights these gave into psychotic experience. He was nevertheless cautious about assuming how much one could interpret what a particular artwork could say about an individual, and certainly refrained from using art as a tool of diagnosis. This raises the epistemological conundrum of how psychotherapy – and indeed other fields of medical and scientific knowledge – make use of the ‘case’, both in its own terms and as an object, or series of objects, from which to develop wider theoretical claims (Forrester, 2016). In this instance, it invites us to reflect on how the study of psychotherapeutic cases can come to generate models of mind and dysfunction, which in turn may shape innovations or modifications in practice.

Ulrich Koch’s article on analytic abstinence and the therapeutic relationship draws our attention to the long-running theoretical debates that can thread through psychotherapeutic professions, in this case over the course of more than a century. Koch also, importantly, brings to the foreground the ways in which these debates from within psychotherapy can have a life in wider cultural arenas. The article shows how they were taken up to inform narratives about cultural decline and narcissism in the postwar western world, in the works of social theorists such as the Frankfurt School and Christopher Lasch.

Finally, Patrick Kirkham’s article charts the invention of Applied Behavioural Analysis (ABA) for autism, its origins in behaviourism and operant conditioning-based therapies, and the controversies that have abounded as a result of its implementation up to the present day. Kirkham describes how many autistic self-advocates have come to eschew the validity of ABA as a therapeutic intervention altogether, alleging that it is rather a form of abuse. Autism is presented by these authors as an indissoluble aspect of identity, and for many as an ontological fact, which demands societal recognition. Attempts to effect a change in, or cure, autistic behaviours ‘therapeutically’ have, ergo, been understood as abusive, all the more so given ABA’s use of aversives. Autistic self-advocates who associate themselves with the neurodiversity movement therefore resist the ‘optional ontology’ of behaviourism, and mobilize instead a language which borrows from neuroscience and cognitive psychology in order to defend an alternative model of mind, one that coheres better with their self-experience.^9^

The new contributions offered here remind us that ‘psychotherapy’ has come to describe many and varied practices since the birth of the term in the 19th century, which are far more diverse than its existing historiography would suggest. It opens up new ways of conceiving the relationalities that underpin therapeutic technique, from Tuke’s assertion that the physician–patient interaction was the key to enabling treatment, to vociferous arguments about therapeutic abstinence and neutrality up to the present. It brings into relief the multiple and competing sites around which the therapeutic ‘cure’ has been located, from suggestion via the ‘imagination’, to patient subordination, to psychodynamic transference or creative expression, through to the aversive conditioning of behaviour. It also raises questions about the different actors involved in the history of psychotherapy, from the obvious candidates of the practitioners themselves and the recipients of their treatments, but also, in some cases, parents and carers, charities and state institutions, scientific researchers, and the place of private practice. Given the multiple uses to which the history of psychotherapy has already been put by clinicians and critics alike, there is an imperative for historians to join in the debate. This special issue hopes to make such an intervention by uncovering therapeutic approaches and the actors who shaped them, disentangling the ethical and epistemological debates that surround their usage, and tracing their cultural imprint.

## Notes

This special issue originated from a conference on Histories of Psychotherapy, organized with Sonu Shamdasani at UCL in October 2013, and I am grateful to him, along with members of the UCL Health Humanities Centre, for discussions over many years. It also owes a debt to the students and staff who attended the Psychotherapy in Historical Perspective seminar at the Department of History and Philosophy of Science, University of Cambridge in 2016: most especially to my co-convener Matthew Drage, and to the late John Forrester for bringing us together for it. Thanks also go to the journal editors, particularly Felicity Callard and Sarah Thompson; and to Daniel Pick.

1. According to survey data collected by *Psychology Today* in 2004: …more than 27% of all adults (an estimated 59 million people) received mental health treatment in the two years prior. Of this group, ‘47% report a history of medication, but no therapy; more than a third (34%) report a history of both medication and therapy; and 19% report a history of therapy, but no medication.’…that means somewhere around 30 million adults were in psychotherapy during that two year period. (Howes, 2008)

2. This literature is too wide-ranging to be fully represented, but a brief selection includes Falzeder (2015); ffytche and Pick (2016); Forrester (1997); Hale (1995); Makari (2008); Plotkin and Damousi (2012); Zaretsky (2005).

3. Rogers has, nevertheless, been the subject of a number of biographies, and participated in the publication of a substantial oral history account of his work. Humanistic psychotherapy has probably, therefore, become one of the fields to have received the most reflective historical attention from psychotherapists themselves, outside of psychoanalysis. See Cohen (1997); Kirschenbaum (1979); Moss (1999); Rogers and Russell (2002).

4. The one potential exception, Martin Liebscher’s article on the Jungian community in Mandate Palestine, latterly Israel, could be argued to be an example of an extension of ‘European’ debates, given the circumstances of recent exile and the continued correspondence with – and deference towards – colleagues on the Continent.

5. Dryden and Still themselves are both academic psychologists and practising psychotherapists, with Dryden having collaborated directly with Ellis (Marks, 2012).

6. For more on moral treatment see Digby (1985); Gauchet and Swain (1994).

7. See also Shamdasani (2010).

8. See also Caplan (2001); Ellenberger (1970); Shamdasani (2012). Outside of the anglophone world, key contributions have been made on modern Japanese therapy, sex and marital therapies in Czechoslovakia, and early psychotherapeutics in Germany. See Harding, Fumiaka and Shin’ichi (2014); Lišková (2016); Knote (2015).

9. Ian Hacking has discussed the ways in which autistic self-advocates have created new languages of human kinds to define their identity, although he does not discuss their engagement with therapeutic interventions (see Hacking, 2009).
